# Universal bound on sampling bosons in linear optics and its computational implications

**DOI:** 10.1093/nsr/nwz048

**Published:** 2019-04-09

**Authors:** Man-Hong Yung, Xun Gao, Joonsuk Huh

**Affiliations:** 1 Shenzhen Institute for Quantum Science and Engineering and Department of Physics, Southern University of Science and Technology, Shenzhen 518055, China; 2 Shenzhen Key Laboratory of Quantum Science and Engineering, Southern University of Science and Technology, Shenzhen 518055, China; 3 Central Research Institute, Huawei Technologies, Shenzhen 518129, China; 4 Center for Quantum Information, Institute for Interdisciplinary Information Sciences, Tsinghua University, Beijing 100084, China; 5 Department of Chemistry, Sungkyunkwan University, Suwon 440-746, Korea

**Keywords:** boson sampling, quantum optics, quantum supremacy, linear optics, computational complexity

## Abstract

In linear optics, photons are scattered in a network through passive optical elements including beam splitters and phase shifters, leading to many intriguing applications in physics, such as Mach–Zehnder interferometry, the Hong–Ou–Mandel effect, and tests of fundamental quantum mechanics. Here we present the fundamental limit in the transition amplitudes of bosons, applicable to all physical linear optical networks. Apart from boson sampling, this transition bound results in many other interesting applications, including behaviors of Bose–Einstein condensates (BEC) in optical networks, counterparts of Hong–Ou–Mandel effects for multiple photons, and approximating permanents of matrices. In addition, this general bound implies the existence of a polynomial-time randomized algorithm for estimating the transition amplitudes of bosons, which represents a solution to an open problem raised by Aaronson and Hance (*Quantum Inf Comput* 2012; **14**: 541–59). Consequently, this bound implies that computational decision problems encoded in linear optics, prepared and detected in the Fock basis, can be solved efficiently by classical computers within additive errors. Furthermore, our result also leads to a classical sampling algorithm that can be applied to calculate the many-body wave functions and the S-matrix of bosonic particles.

## INTRODUCTION

Apart from being of fundamental interest in physics, linear optics has become a simple but powerful tool for processing quantum information [1–5] and quantum simulation [6–11]. One of the major advantages for encoding information with light is that photons are highly robust against decoherence, which makes it an ideal system to study quantum coherence [12–16]. Furthermore, in linear optical networks, all possible transformations can be achieved with simple operations involving at most a pair of modes; more precisely, every optical circuit can be implemented with beam splitters and phase shifters only [17]. Linear optical networks have been routinely built in photonic chips [18–20] using standard semiconductor fabrication technology. In particular, a reprogrammable linear optical circuit has been integrated into a photonic chip [20], which can perform universal operations on six photonic modes with up to six photons. In addition, photonic chips can also be applied for demonstrating different physics [21,22].

What about the computational power of linear optics? Knill, Laflamme and Milburn (KLM) [23] proved a universality theorem showing that linear optics, together with post-selected measurements, is powerful enough for universal quantum computation. Aaronson adopted this fact to prove that computation of the permanent of a matrix is #P hard. However, can linear optics outperform a classical device in some computational problems, even without adaptive measurements? This is the main question that we address here.

**Figure 1.**
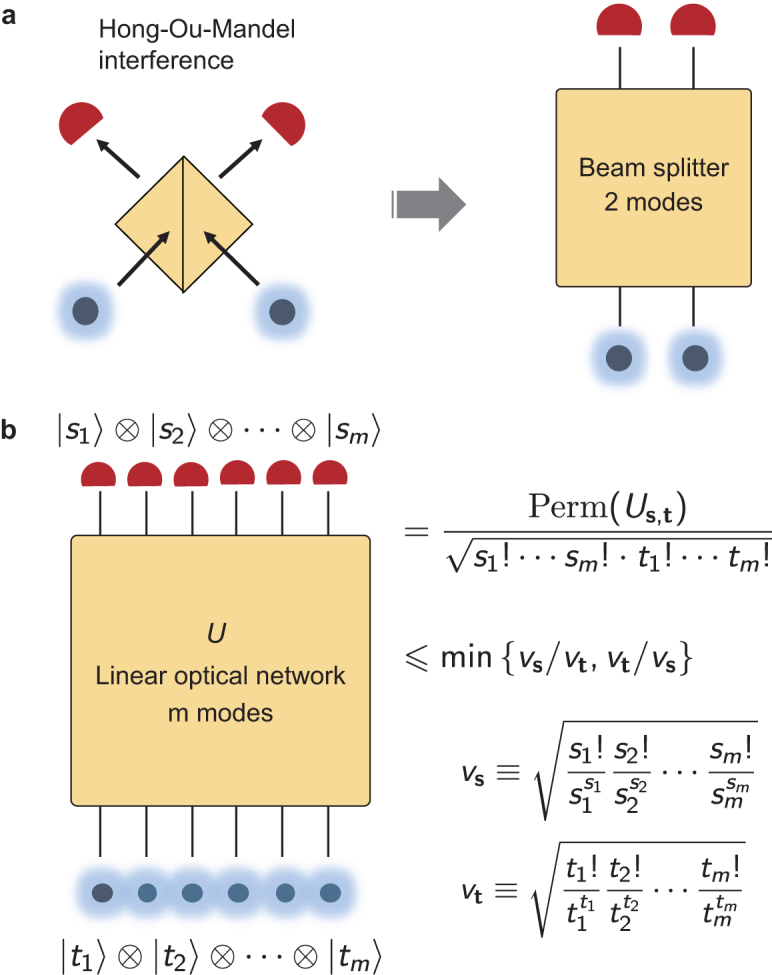
(a) The Hong–Ou–Mandel interference can be viewed as a special instance of boson sampling, where the number of modes is two, and the linear network contains a 50:50 beam splitter only. (b) Summary of our main result: an upper bound of the transition amplitudes for linear optics. The initial and final states are products of Fock states. The matrix *U* presents any realizable unitary transformation in linear optics.

Recently, it was found that boson sampling [24–28], as a novel application of linear optics, can be regarded as evidence for proving the inefficiency of classical devices to perform quantum simulation, which represents a serious challenge to the validity of the extended Church–Turing thesis [1]. Furthermore, a special type of correlation, called forrelation [29,30] has been identified as being able to exhibit the largest possible separation between quantum and classical query complexities. In boson sampling [24], a product of single-photon states is injected into a linear photonic network that encodes an instance of complex matrices. In fact, the famous Hong–Ou–Mandel interference can be viewed as a special instance of boson sampling (see Fig. 1). The ability to approximate the corresponding permanents of matrices with a multiplicative error implies the ability of simulating boson sampling, which is widely believed to be impossible, based on computational complexity assumptions [24]. With this motivation, much progress [20,31–36] has been made in the experimental realization of boson samplers using linear optical devices.

Specifically, the problem of interest in this work is described as follows: let us suppose that we are given a product of Fock states containing a total of *n* identical photons (or generally bosons) distributed over *m* different modes, i.e.

(1) \begin{equation*} \left| {{t_1}{t_2} \cdots {t_m}} \right\rangle \equiv \left| {{t_1}} \right\rangle \otimes \left| {{t_2}} \right\rangle \otimes \cdots \otimes \left| {{t_m}} \right\rangle , \end{equation*}

where $\left| {{t_k}} \right\rangle \equiv {({t_k}!)^{ - 1/2}}{(a_k^\dagger )^{{t_k}}}\left| {{\text{vac}}} \right\rangle$ contains *t_k_* photons for *t_k_* = 0, 1, 2, …, *n*. Moreover, the state is subject to the constraint of particle conservation:

(2) \begin{equation*} \sum \limits_{k = 1}^m {{t_k}} = n. \end{equation*}

Here $a_k^{\dagger }$ creates a boson in *k*th mode and satisfies the commutation relations ${}[{a_j},a_k^\dagger ] \equiv {a_j}a_k^\dagger - a_k^\dagger {a_j} = {\delta _{ij}}$, ${}[a_j^\dagger ,a_k^\dagger ] = [{a_j},{a_k}] = 0$.

Let us consider *any* member *U* in the set of all possible unitary operators (i.e. linear optics) that induces a linear transformation (i.e. non-interacting) for the boson modes, i.e.

(3) \begin{equation*} Ua_k^\dagger {U^\dagger } = \sum \limits _{j = 1}^m {{u_{kj}}} a_j^\dagger . \end{equation*}

The central problem is to give an upper bound for the absolute value of the transition amplitude, |〈*s*_1_*s*_2_ ⋅ ⋅ ⋅ *s_m_*|*U*|*t*_1_*t*_2_ ⋅ ⋅ ⋅ *t_m_*〉|, for locating the resulting state in another given product state, |*s*_1_*s*_2_ ⋅ ⋅ ⋅ *s_m_*〉, subject to the same particle-conserving constraint, $\sum \nolimits _{k = 1}^m {{s_k}} = n$, for *s_k_* = 0, 1, 2, …, *n*. This problem is solved in this work, where an explicit form of the bound is given in Eq. (4), which limits the efficiency of sampling bosons for all possible linear optical networks, including the behaviors of Bose–Einstein condensates (BEC) in linear networks, and the counterparts of Hong–Ou–Mandel effects for multiple photons. Furthermore, this bound is important for our proof on the existence of a polynomial-time randomized algorithm for approximating permanents of matrices, which represents a solution to an open problem posed by Aaronson and Hance [37].

In terms of computational complexity, this algorithm establishes that the computational complexity class of decision problems encoded in an optical network cannot exhibit quantum supremacy [38], as it can be solved by a classical randomized algorithm. Previously, it was known that a quantum computational model with Clifford gates [39], sparse distribution [40], and fermionic (matchgates) [41–43] can be simulated by classical computers. Our result completes the picture by showing that computational decision problems encoded with non-interacting bosons can also be simulated classically (but sampling bosons remain a hard problem under computational-theoretic assumptions).

We remark that our results are built on the existing results of Aaronson and Hance [37], who have already demonstrated an efficient classical algorithm for a special case where the initial state is confined to be those with either 1 or 0, e.g. |11 ⋅ ⋅ ⋅ 10 ⋅ ⋅ ⋅ 0〉, but not for the states with one or more photons, e.g. |22 ⋅ ⋅ ⋅ 20 ⋅ ⋅ ⋅ 0〉 or |*n*00 ⋅ ⋅ ⋅ 0〉 for *n* ≥ 2. In addition, Aaronson and Hance posed an open question [37] asking whether it is possible to extend the result to the cases where the initial state can be an arbitrary Fock product state, i.e. |*t*_1_*t*_2_ ⋅ ⋅ ⋅ *t_m_*〉. In this work, we adopted a different mathematical technique to achieve the goal of constructing such a classical algorithm and answering the open question positively. However, this does not imply that linear optical devices cannot perform hard computational problems, but it does have implications for decision problems (which are discussed later in the paper).

In practice, such a classical algorithm can be applied to calculate the many-body wave functions and the S-matrix of bosonic particles; this is made possible by a technique called ‘operator-to-number conversion’ developed in this work. In the online supplementary material, we provide a compact introduction to various concepts related to this work.

## RESULTS

### Statement of the main result

We shall prove that the upper bound of the boson transition amplitude is given by the following expression:

(4) \begin{equation*} \left| {\langle {s_1} \cdots {s_m}|U\left| {{t_1} \cdots {t_m}} \right\rangle } \right| \le \min \left\lbrace {{v_{s}}/{v_{t}},{v_{t}}/{v_{s}}} \right\rbrace , \end{equation*}

where $v_{s}$ is a product of *m* factors generated from the elements in the list ${s}=(s_1,s_2,\ldots ,s_m)$,

(5) \begin{equation*} {v_{s}} \equiv \sqrt{({s_1}!/s_1^{{s_1}})({s_2}!/s_2^{{s_2}}) \cdots ({s_m}!/s_m^{{s_m}})} , \end{equation*}

and defined similarly for $v_{t}$ (see Fig. 1). If one of the modes is unoccupied, e.g. *s_k_* = 0, then we simply set ${s_k}!/s_k^{{s_k}} \rightarrow 1$.

An immediate consequence of our bound is that a necessary condition for a *perfect* transition from a general Fock state to another Fock state is that ${v_{s}} = {v_{t}}$.

In the context of boson sampling [24], the initial state is always a product of single-photon states, i.e. |*t*_1_*t*_2_ ⋅ ⋅ ⋅ *t_m_*〉 = |111 ⋅ ⋅ ⋅ 00〉. In this case, we can recover the result obtained previously by Aaronson and Hance [37], which is a special case of our result, i.e. $\left| {\left\langle {{s_1}{s_2} \cdots {s_m}} \right|U\left| {111 \cdots 00} \right\rangle } \right| \le {v_{s}}$.

Before we go into the details of the proof of the bound, we first discuss the physical and computational implications of the bound.

### Absence of exact boson bunching

If we further set *s*_1_ = *n*, and *s*_2_ = *s*_3_ ⋅ ⋅ ⋅  = *s_m_* = 0, the probability of putting all bosons into the same mode from |111 ⋅ ⋅ ⋅ 00〉 is exponentially low, as (previously obtained in Ref. [37])

(6) \begin{eqnarray*} {P_{\max }}\left( {n,0\cdots 0|1,1\cdots 1} \right) &=& {v_{s}^2} = {n!/{n^n}}\nonumber\\ & \approx& \sqrt{2\pi n} \, {e^{ - n}} , \end{eqnarray*}

using the Stirling approximation, $n! \approx \sqrt{2\pi n} \, {\left( {n/e} \right)^n}$. Consequently, for *n* ≥ 3,

(7) \begin{equation*} {P_{\max }}\left( {3,0,0|1,1,1} \right) = 3!/{3^3} = 2/9 ; \end{equation*}

one cannot observe the generalization of the Hong–Ou–Mandel effect with linear optics, i.e. going from the state |11 ⋅ ⋅ ⋅ 1〉 to |*n*0 ⋅ ⋅ ⋅ 〉 + |0*n*0 ⋅ ⋅ ⋅ 〉 + |00*n*0 ⋅ ⋅ ⋅ 〉; the reason that the Hong–Ou–Mandel effect is possible for the case of *n* = 2 is because the bound is given by

(8) \begin{equation*} {P_{\max }}\left( {2,0|1,1} \right) = v_{s}^2 = 2!/{2^2} = 1/2 , \end{equation*}

but there are two modes; the total probability can therefore reach unity. This result is complementary to a previous result [44] showing the absence of the Hong–Ou–Mandel dip with the Bell-multiport beam splitter.

### Boson bunching limits

Furthermore, we can find the upper limit on the transition probabilities in general scenarios. For example, imagine that there are *p* bosons in one mode and *q* bosons in another mode. Suppose that we are interested in the case where all bosons are grouped into a single mode, i.e.

(9) \begin{equation*} {( {{a^\dagger _1 }} )^p}{( {{a^\dagger _2 }} )^q}\left| {{\text{vacuum}}} \right\rangle \rightarrow {( {{a^\dagger _1 }} )^{p + q}}\left| {{\text{vacuum}}} \right\rangle . \end{equation*}

From Eq. (4), the probability of getting *p* + *q* in a single mode through linear optics is then bounded by the following:

(10) \begin{equation*} {P_{\max }}\left( {p + q,0|p,q} \right) = \frac{{(p + q)!}}{{p!q!}}\frac{{{p^p}{q^q}}}{{{{(p + q)}^{p + q}}}} , \end{equation*}

or its inverse. As far as we are aware, this bound is new in quantum optics. To be specific, let us consider the following cases.

### Case 1: creation of a mode with 2*n* bosons from two separate modes with *n* bosons each

In this case, we have

(11) \begin{equation*} {P_{\max }}\left( {2n|n,n} \right) = 2n!/n{!^2}{2^{2n}} . \end{equation*}

In the limit of a Bose–Einstein condensate (BEC), meaning a very large Fock state |*n*〉 with *n* ≫ 1, the probability bound

(12) \begin{equation*} {P_{\max }}\left( {2n|n,n} \right) \approx 1/\sqrt{\pi n} \end{equation*}

decreases as $O(1/\sqrt{n})$. An optimal strategy for achieving the bound is to apply the 50:50 beam splitter, i.e. $a_1^\dagger \rightarrow (a_1^\dagger + a_2^\dagger )/\sqrt{2}$ and $a_2^\dagger \rightarrow (a_1^\dagger - a_2^\dagger )/\sqrt{2}$. Note that the reverse process, i.e. splitting a BEC into halves, is equally inefficient with linear optics.

### Case 2: adding one extra boson to a BEC using linear operations

Supposing that *p* = *n* and *q* = 1, the bound is given by

(13) \begin{equation*} {P_{\max }}\left( { n+1,0|n,1} \right) = {\left( {n/(n + 1)} \right)^n} , \end{equation*}

which approaches a constant limit, *e*^−1^, when *n* → ∞. In fact, this bound can be saturated by the following transformation: $U_n a_1^\dagger {U_n^\dagger } = \cos \theta _n \, a_1^\dagger + \sin \theta _n \, a_2^\dagger$, where sin ^2^θ_*n*_ = (*n* + 1)^−1^.

### Absence of deterministic boson adders

Note that the process of boson bunching as described in Eq. (9) can be viewed as a process of arithmetic addition using bosons. These results impose limitations to the efficiency of performing arithmetic operations using pure linear optics, which is necessarily a probabilistic process as we have seen, unless extra degrees of freedom are allowed (see e.g. Ref. [45]).

### Quantum superposition ≠ classically intractable

By showing that classical algorithms can solve the class of decision problems of sampling bosons, we can establish the following fact: although the key feature of quantum computation comes from the ability to create a superposition of an exponential number of states, our results provide explicit evidence that this quantum ability cannot be a sufficient condition for exhibiting quantum advantage in computational tasks over classical devices. Similar conclusions can be achieved for other quantum computing models, such as with Clifford gates [39], sparse distribution [40], and fermionic (matchgates) [41–43]. Our result is complementary to these models in the context of linear optics.

In other words, non-classical decision problems in linear optics, assuming that they are encoded in polynomially many output states, can be solved.

### Bounding size of matrix permanents

Another implication of our main result is related to permanents of matrices. The transition amplitude in Eq. (4) is known (see e.g. Ref. [24]) to be related to a permanent of a matrix regarding the unitary operator *U*:

(14) \begin{eqnarray*} &&\left\langle {{s_1}{s_2}\cdots {s_m}} \right|U\left| {{t_1}{t_2}\cdots {t_m}} \right\rangle\nonumber\\ &&\quad = \frac{{{\text{Perm}}\left( {{U_{{s},{t}}}} \right)}}{{\sqrt{{s_1}! \cdots {s_m}! \cdot {t_1}! \cdots {t_m}!} }} , \end{eqnarray*}

where $U_{{s},{t}}$ is an *n* × *n* matrix constructed by the transformation elements *u_kj_* (see Eq. (3)) of the unitary operator *U* in the following way: create *s_k_* copies of a row of vectors ${\mu }_{k,{t}}$ that contain *t_j_* copies of *u_kj_*. The special case where all *t_i_* ∈ {0, 1} was discussed in Ref. [37].

For example, if ${s} = \left( {1,0,2} \right)$ and ${t} = \left( {2,1,0} \right)$, then the matrix $U_{{s},{t}}$ is of the following form:

(15) \begin{equation*} {U_{{s},{t}}} = \left[ {\begin{array}{*{20}{c}}{{\mu } _{1,{t}}} \\ {{{\mu } _{3,{t}}}}\\ {{{\mu } _{3,{t}}}}\end{array}}\right] = \left[ {\begin{array}{c@{\quad}c@{\quad}c} {u_{1,1}}&{{u_{1,1}}}&{{u_{1,2}}} \\ {{u_{3,1}}}&{{u_{3,1}}}&{{u_{3,2}}} \\ {{u_{3,1}}}&{{u_{3,1}}}&{{u_{3,2}}} \end{array}} \right]. \end{equation*}

Note that if all *s* and *t* equal unity, then the transition probability is exactly the same as the permanent of the matrix defined in Eq. (3), i.e.

(16) \begin{equation*} \langle 11\cdots 1|U\left| {11\cdots 1} \right\rangle = {\text{Perm}}(u_{kj}) . \end{equation*}

In other words, our bound also implies an upper bound of the permanent of a matrix:

(17) \begin{equation*} | \, {\text{Perm}}({U_{{s},{t}}}) \, | \le \min \left\lbrace {\frac{{{v_{s}}}}{{{v_{t}}}},\frac{{{v_{t}}}}{{{v_{s}}}}} \right\rbrace \times \prod \limits _{k = 1}^m {\sqrt{{s_k}! \, {t_k}!} } . \end{equation*}

### Efficient representation and calculations of many-body wave functions

As a further application, we have developed a new operator-to-number technique, which can be employed to represent bosonic many-body wave functions,

(18) \begin{equation*} \phi ( {n}) \equiv \big\langle {n} \big|\psi \big( {a_1^\dagger ,a_2^\dagger ,\ldots ,a_n^\dagger } \big)\big| {{\text{vac}}} \big\rangle , \end{equation*}

in terms of a summation of complex numbers (see Eq. (21)). Moreover, the many-body wave function $\phi \left( {n} \right)$ can be estimated efficiently by a random sampling of the complex numbers (see Eq. (23)).

Additionally, the S-matrix in quantum field theory can be reduced to a problem of calculating the permanents of certain matrices [46], which can also be solved by our classical randomized algorithm in polynomial time.

### Implication of the open problem on computational complexity

Before this work, it was not known if it was possible to create classically hard ‘decision problems’ with linear quantum optics that involve the determination of the transition amplitudes. One of the major questions in the field of linear optics is whether boson sampling can be extended to solving decisions problems, such as factoring, etc., within additive errors.

Now, the existence of the classical randomized algorithm presented in this work implies that (S. Aaronson, private communication) any decision problem involving only one (or a polynomial number) of boson transition amplitudes can be solved efficiently with a classical device, in the context of computational complexity theory. To be more specific, the open problem [37] raised by Aaronson and Hance asks,

Can we estimate any linear-optical amplitude (see Eq. (14)) to ±1/poly(*n*) additive error (or better) in polynomial time?

With our bound shown in Eq. (4), we confirm that there does exist a polynomial-time randomized algorithm for the general cases. Thus, the open problem is now settled for those decision problems encoded in (a polynomial number of) the transition amplitudes.

### Decision problems of boson sampling

Recall that we are dealing with decision problems instead of sampling problems for linear optics. The sampling problems require an estimation of the transition amplitude to a multiplicative error, or equivalently the ability to reproduce the target distribution, i.e. weak sampling. For decision problems, one is required to estimate the probability directly, i.e. strong simulation (see e.g. Refs. [47,48] for a further discussion on the relationship between strong and weak simulation).

To elaborate further, recall the definition of the complex class BQP (bounded-error quantum polynomial time), which represents the class of languages that can be decided with high probability by polynomial-size uniform quantum circuit families. More precisely, a language *L* is in BQP if and only if there exists a polynomial-time uniform family of quantum circuits *Q_n_*(*x*), which takes *n* qubits and outputs 1 bit, such that

1. for all *x* ∈ *L*, $\Pr (Q_n(x)=1) \ge 2/3$,
2. for all *x* ∉ *L*, $\Pr (Q_n(x)=1) \le 1/3$.

For example, one may start with the all-zero state, |0^⊗*n*^〉 ≡ |000 ⋅ ⋅ ⋅ 0〉. The quantum circuit associated with the instance *x* can be represented by a unitary transformation *U_x_*. The quantum circuit may accept when one of the outcomes, e.g. |*y*〉, appears. In other words, we may write

(19) \begin{equation*} \Pr ( {{Q_n}( x ) = 1} ) = {| {\langle y |{U_x}| {{0^{ \otimes n}}} \rangle } |^2} . \end{equation*}

In our case, we replace the quantum circuit in BQP with a linear optical network $U_x^{{\text{LO}}}$, which is a subset of BQP, for a fixed number of bosons. However, we consider not only a fixed initial state, but any arbitrary Fock state |*t*_1_*t*_2_ ⋅ ⋅ ⋅ *t_m_*〉 of *n* photons, which means that a decision problem encoded in linear quantum optics can be formulated as follows:

(20) \begin{eqnarray*} &&\Pr ( {Q_m^{{\text{LO}}}( x ) = 1} )\nonumber\\ &&\quad =\, {| {\langle {{s_1}{s_2} \cdots {s_m}} |U_x^{{\text{LO}}}| {{t_1}{t_2} \cdots {t_m}} \rangle } |^2} , \end{eqnarray*}

where ${Q_m^{{\text{LO}}}}$ denotes a linear optical network of *m* modes.

Note that an alternative definition of decision problems for boson sampling has been defined [49]. There, decision problems of boson sampling are defined as problems to decide if a function *f*(*x, y*) satisfies a certain property. Here *x* is generated by the most probable bin (MPB) of a boson-sampling instance, and *y* is an optional string. In other words, by grouping the outcomes of boson sampling into bins, one is required to find the bin associated with the largest probability.

In fact, if we can calculate the individual transition amplitude (i.e. Eq. (14)) accurately, then we can also find such an MPB by generating a probability distribution that approximates the original distribution (see Refs. [49–51]).

**Figure 2.**
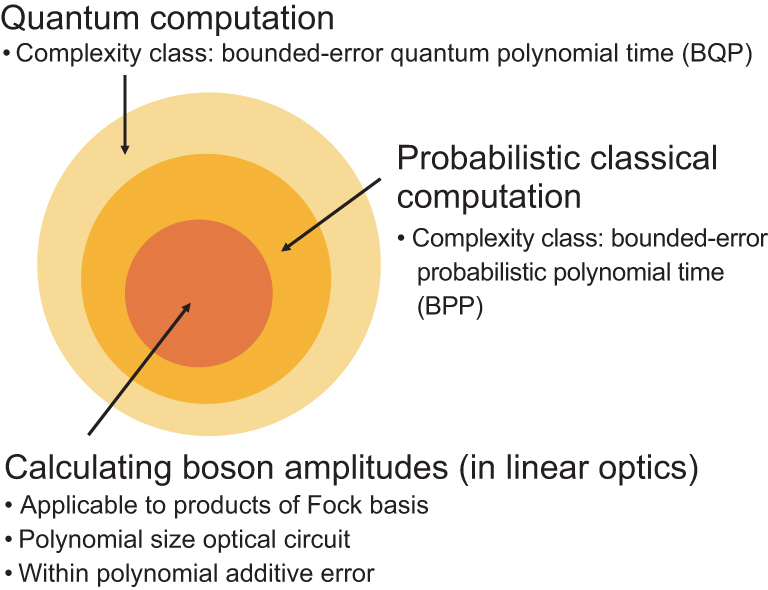
Relationship between the complexity class of estimating boson amplitude, and classical and quantum computation. Our result establishes that calculating the boson amplitude, with a polynomial additive error, is a problem inside BPP.

Given the existence of the polynomial-time classical algorithm in this work, which can estimate boson transition amplitude within additive error, conditions (i) and (ii) can be readily satisfied with a classical computer, which means that they are actually problems inside the complexity class BPP (see Fig. 2). Finally, we note that our classical algorithm is designed to estimate the amplitude up to an additive error. However, to solve the sampling problem of linear optics, i.e. boson sampling where the amplitudes become exponentially small, it will require the estimation of the amplitudes to within multiplicative errors. As a result, our algorithm will need to run for an exponential time to estimate the amplitudes to within multiplicative errors.

Overall, our result imposes a new constraint for obtaining quantum computational supremacy with linear optics over classical computers, in the context of solving decision problems.

## DERIVATION OF MAIN RESULTS

**Figure 3.**
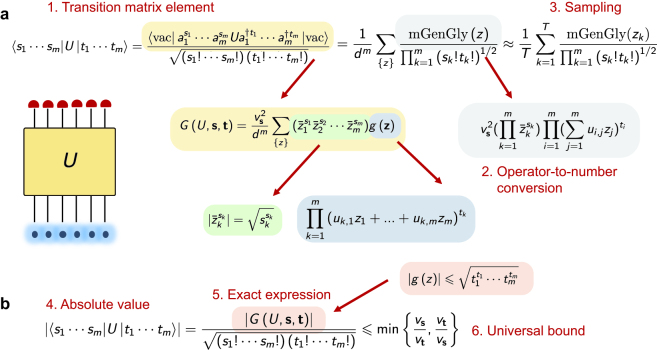
Summary of the relationships with the transition amplitude. (a) The transition matrix element can be transformed into a sum of complex variables, through the operator-to-number conversion technique. (b) The absolute value of the transition can be bounded by calculating $v_{s}$ and $v_{t}$.

Let us now establish a general theorem that is crucial for our result (see Fig. 3 for a summary). Similar to Feynman’s path integrals, this theorem tells us how to perform operator-to-number conversion for bosonic transition amplitudes. However, unlike Feynman’s path integrals, the number of variables depends on the number of modes, instead of infinite-dimensional integrals, and there is no sign problem.

### Generalized transition amplitude

Given any polynomial function, $f(a_1^\dagger ,a_2^\dagger ,\ldots ,a_m^\dagger )$, of multi-mode creation operators $a_k^\dagger$, the vacuum-to-vacuum transition amplitude (unnormalized),

(21) \begin{equation*} {F_{s}}\,{ \equiv}\, \big\langle {\rm vac} \big|a_1^{{s_1}}a_2^{{s_2}} \cdots a_m^{{s_m}} \, f\big(a_1^\dagger ,a_2^\dagger ,\ldots ,a_m^\dagger\big )\big| {\rm vac} \big\rangle , \end{equation*}

can always be expressed as a sum involving a set of weighted complex roots of unity, by mapping the boson operator,

(22) \begin{equation*} {a_k^\dagger } \ \rightarrow \ {z_k} , \end{equation*}

to a complex number *z_k_*, and similarly $a_k \rightarrow {{\bar{z}}_k}$ to its complex conjugate ${\bar{z}}_k$:

(23) \begin{equation*} {F_{s}} = \frac{v_{s}^2}{{{d^m}}}\sum \limits _{\lbrace {z}\rbrace } {{\bar{z}}_1^{{s}_1}} {{\bar{z}}_2^{{s_2}} \cdots {\bar{z}}_m^{{s_m}} \, f({{ z}_1},{{ z}_2},\ldots ,{{z}_m})} , \end{equation*}

where

(24) \begin{equation*} {z_j} \in \big\lbrace \sqrt{{s_j}} \, { \omega ^0}, \sqrt{{s_j}} \, {\omega ^1},\ldots , \sqrt{{s_j}} \, {\omega ^{d - 1}}\big\rbrace \end{equation*}

is related to one of the complex roots of unity ω ≡ *e*^−2π*i*/*d*^, weighted by a factor $\sqrt{{s_j}}$. Here *d* is chosen to be an integer larger than the degree of the function and the sum $\sum \nolimits _{k = 1}^m {{s_k}}$.

Alternatively, we can write $F_{s}$ in the form of an expectation value:

(25) \begin{equation*} {F_{s}} = {v_{s}^2} \, {\mathbb {E}} \big[\bar{z}_1^{{s_1}}\bar{z}_2^{{s_2}} \cdots \bar{z}_m^{{s_m}}f({z_1},{z_2},\ldots ,{z_m})\big] , \end{equation*}

which allows us to devise a sampling algorithm to estimate its value, as we shall discuss later.

### Technique of operator-to-number conversion

Here we introduce a technique of operator-to-number conversion. Since all the terms in the function $f(a_1^\dagger ,a_2^\dagger ,\ldots ,a_m^\dagger )$ commute with one another, we can e.g. sort out the first creation operator $a_1^{\dagger }$ as if it was just a real number, and write

(26) \begin{equation*} f(a_1^\dagger ,a_2^\dagger ,\ldots ,a_m^\dagger ) = \sum \limits _{k = 0}^d {a_1^{\dagger k}{\phi _k}(a_2^\dagger ,\ldots ,a_m^\dagger )} , \end{equation*}

where ${{\phi _k}(a_2^\dagger ,\ldots ,a_m^\dagger )}$ is a resulting polynomial function without $a_1^\dagger$. Consequently, we have

(27) \begin{eqnarray*} {F_s} &=& \sum \limits _{k = 0}^d {\big\langle {\text{0}} |a_1^{{s_1}}} a_1^{\dagger k}| {\text{0}} \big\rangle\nonumber\\ && \big\langle {{\text{vac}}} \big|a_2^{{s_2}} \cdots a_m^{{s_m}} \, {\phi _k}\big(a_2^\dagger ,\ldots ,a_m^\dagger\big )\big| {{\text{vac}}} \big\rangle .\nonumber\\ \end{eqnarray*}

Note that there is only one non-zero term in the summation, as

(28) \begin{equation*} \big\langle {\text{0}} \big|a_1^{{s_1}}a_1^{\dagger k}\big| {\text{0}} \big\rangle = s_1! \, {\delta _{{s_1}k}} . \end{equation*}

Now, since the Kronecker delta function can be expressed (by the representation through discrete Fourier transform) as follows: ${\delta _{{s_1}k}} = ( {1/d} )\sum \nolimits _{j = 0}^{d - 1} {{e^{ - ( {2\pi ij/d} )({s_1} - k)}}} = ( {1/d} )\sum \nolimits _{j = 0}^{d - 1} {{\omega ^{j(k-{s_1})}}}$, we can therefore write the inner product (with $z_1 \in \lbrace \sqrt{s_1} \omega ^0, \sqrt{s_1} \omega ^1, \ldots , \sqrt{s_1} \omega ^{d-1} \rbrace$),

(29) \begin{equation*} \big\langle {\text{0}} \big|a_1^{{s_1}}a_1^{\dagger k}\big| {\text{0}} \big\rangle = \frac{s_1!}{s_1^{s_1}} \frac{1}{d}\sum \limits _{\lbrace {z_1}\rbrace } {{\bar{z}}_1^{{s_1}} z_1^k} , \end{equation*}

as a sum over all values of *z*_1_, which implies that we can replace the operators with complex numbers, i.e. ${F_s} = ({s_1}!/s_1^{{s_1}}d)\sum \nolimits _{\lbrace {z_1}\rbrace } {{\bar{z}}_1^{{s_1}}\left\langle {\rm vac} \right|a_2^{{s_2}} \cdots a_m^{{s_m}}f({{ z}_1},a_2^\dagger ,\ldots ,a_m^\dagger )\left|\, {\rm vac} \right\rangle}$. Next, we can define a new polynomial function,

(30) \begin{eqnarray*} &&f^{\prime }(a_2^\dagger ,\ldots ,a_m^\dagger ) \equiv \left( {s_1!/s_1^{s_1} d} \right)\nonumber\\ &&\quad\times\,\sum \limits _{\lbrace {z_1}\rbrace } {{\bar{z}}_1^{{s_1}}f({{ z}_1},a_2^\dagger ,\ldots ,a_m^\dagger )} , \end{eqnarray*}

and repeat the same procedure for $a_2^{\dagger }$, and so on, which yields the result in Eq. (23) at the end.

### Three-step derivation of the universal bound

We are now ready to present the proof for the bound in Eq. (4). For this purpose, we express the transition amplitude explicitly with bonsonic operators, i.e.

(31) \begin{eqnarray*} &&\langle {s_1} \cdots {s_m}|U\!\left| {{t_1}\cdots {t_m}} \right\rangle\nonumber\\ &&\quad =\, \frac{{G\!\left( {U,{s},{t}} \right)}}{{\sqrt{({s_1}! \cdots {s_m}!)({t_1}! \cdots {t_m}!)} }} , \end{eqnarray*}

where we have defined an operator function:

(32) \begin{eqnarray*} && G\!\left( {U,{s},{t}} \right)\nonumber\\ &&\quad \equiv \big\langle {{\text{vac}}} \big|a_1^{{s_1}} \cdots a_m^{{s_m}} \, U \, a_1^{\dagger {t_1}} \cdots a_m^{\dagger {t_m}}\big| {{\text{vac}}} \big\rangle . \end{eqnarray*}

The proof can be completed with only three steps as follows.

#### Step 1 (operator-to-number conversion)

With the transformation rule given in Eq. (3), we have

(33) \begin{eqnarray*} &&U a_1^{\dagger {t_1}} \cdots a_m^{\dagger {t_m}} U^\dagger\nonumber\\ &&\quad = \prod \nolimits _{k = 1}^m {\big({u_{k,1}}} a_1^\dagger + \cdots + {u_{k,m}}a_m^\dagger {\big)^{{t_k}}} , \end{eqnarray*}

which is exactly a polynomial function of the creation operators. Therefore, the theorem above implies that

(34) \begin{equation*} G\!\left( {U,{s},{t}} \right) = \frac{{{v_{s}^2}}}{{{d^m}}}\sum \limits _{\lbrace {z} \rbrace } \big(\bar{z}_1^{{s_1}}\bar{z}_2^{{s_2}} \cdots \bar{z}_m^{{s_m}}\big) \, {g( {z})} , \end{equation*}

where the function $g(z)$ is defined as follows:

(35) \begin{equation*} g\!\left({z} \right) \equiv \prod \limits _{k = 1}^m {{{({u_{k,1}}{z_1} + \cdots + {u_{k,m}}{z_m})}^{{t_k}}}} . \end{equation*}

In order to bound the absolute value of $G(U,{s},{t})$, it is sufficient to bound the function $g({z})$ by writing its absolute value in the following form: $\left| {g\!\left({z} \right)} \right| = \sqrt{t_1^{{t_1}}\cdots t_m^{{t_m}}} \prod \nolimits _{j = 1}^m {{{(1/\sqrt{{t_j}} )}^{{t_j}}}} |{u_{k,1}}{z_1} + \cdots + {u_{k.m}}{z_m}{|^{{t_j}}}$.

#### Step 2 (arithmetic–geometric inequality)

Recall that the weighted arithmetic–geometric inequality suggests that

(36) \begin{equation*} A_1^{{\lambda _1}}A_2^{{\lambda _2}} \cdots A_m^{{\lambda _m}} \le {\lambda _1}{A_1} + {\lambda _2}{A_2} + \cdots + {\lambda _m}{A_m} \end{equation*}

for all non-negative *A_k_* and λ_*k*_, subject to the constraint $\sum \nolimits _{k = 1}^m {{\lambda _k}} = 1$. In terms of our *t* (by setting λ_*k*_ = *t_k_*/*n*), we have ${(A_1^{{t_1}}A_2^{{t_2}} \cdots A_n^{{t_n}})^{1/2}}\, \le\, {[({t_1}/n){A_1} + ({t_2}/n){A_2} + \cdots + ({t_n}/n){A_n}]^{n/2}}$. Now, let us denote

(37) \begin{equation*} {A_j} = \left( {1/{t_j}} \right)|{u_{k,1}}{z_1} + \cdots + {u_{k.m}}{z_m}{|^2} . \end{equation*}

Then, we have,

(38) \begin{eqnarray*} &&\frac{{\left| {g\!\left({z} \right)} \right|}}{{\sqrt{t_1^{{t_1}}\cdots t_m^{{t_m}}} }}\nonumber\\ &&\quad \le {\Bigg(\sum \limits _{j = 1}^m {\frac{1}{n}|{u_{k,1}}{z_1} + \cdots + {u_{k.m}}{z_m}{|^2}} \Bigg)^{n/2}}\!\! . \end{eqnarray*}

#### Step 3 (bounding the norms)

Note that the right-hand side is related to the 2-norm of a vector, which is defined by: $\left\Vert {z} \right\Vert \equiv {\left\Vert {z} \right\Vert _2} = \sqrt{|{z_1}{|^2} + |{z_2}{|^2} + \cdots + |{z_m}{|^2}}$. To take a step further, we can always define a unitary matrix *V* such that

(39) \begin{equation*} {( {V{z}})_k} = {u_{k,1}}{z_1} + \cdots + {u_{k,m}}{z_m} , \end{equation*}

which implies that

(40) \begin{equation*} \left| {g\!\left( {z} \right)} \right| \le {\big(t_1^{{t_1}}\cdots t_m^{{t_m}}/n^n\big)^{1/2}}{\left\Vert {V{z}} \right\Vert ^{n/2}} . \end{equation*}

Since ‖*V*‖ = 1 for unitary matrices, and $\left\Vert {z} \right\Vert = {({s_1} + {s_2} + \cdots {s_m})^{1/2}} = \sqrt{n}$, we have

(41) \begin{equation*} \left| {g\!\left( {z} \right)} \right| \le {\big(t_1^{{t_1}}\cdots t_m^{{t_m}}\big)^{1/2}} . \end{equation*}

Consequently, as $| {\bar{z}_k^{{s_k}}} | = s_k^{{s_k/2}}$, we have $| {G\!( {U,{s},{t}} )} | \le ( {v_{s}^2/{d^m}}){( {s_1^{{s_1}} \cdots s_m^{{s_m}}})^{1/2}}\sum \nolimits _{\lbrace {z} \rbrace } {| {g\!( {z} )} |}$, and hence

(42) \begin{equation*} |G\!\left( {U,{s},{t}} \right) |\le v_{s}^2\sqrt{(s_1^{{s_1}} \cdots s_m^{{s_m}})(t_1^{{t_1}} \cdots t_m^{{t_m}})} , \end{equation*}

which implies part of the advertised result of the bound $v_{s}/v_{t}$ in Eq. (4). We can repeat essentially the same procedure for the complex conjugate, 〈*t*_1_ ⋅ ⋅ ⋅ *t_m_*| *U*^†^|*s*_1_ ⋅ ⋅ ⋅ *s_m_*〉, of the transition amplitude, in order to obtain the other part, $v_{s}/v_{t}$. This completes our proof for the bound in Eq. (4).

### Generalized Glynn’s estimators

In Ref. [37], Aaronson and Hance proposed a generalization of Gurvits’s algorithm by defining a generalized Glynn’s estimator, namely

(43) \begin{eqnarray*} {\rm GenGly}({z}) &\equiv& {v_{s}^2} \, \big(\bar{z}_1^{{s_1}} \cdots \bar{z}_m^{{s_m}}\big)\prod \limits _{i = 1}^m\nonumber\\ &&\times {({w_{i,1}}{z_1} + \cdots + } {w_{i,m}}{z_m}) , \end{eqnarray*}

where $v_{s}$ is defined in Eq. (5). Sampling the generalized Glynn’s estimator over the complex values, the permanent, Perm(*V*), of a matrix *V*, which is obtained by repeating *s_i_* times the *i*th row of the *m* × *m* matrix *W* = (*w_i, j_*), can be estimated in polynomial time with an additive error $\pm \, \epsilon \, {v_s} \sqrt{{s_1}! \cdots {s_m}!} \, {\left\Vert W \right\Vert ^n}$. With the new bound, we are able to construct a more general estimator (see Eq. (46)) and bound the size of it. As a result, we are able to estimate matrix permanents and the boson transition amplitude through the relation shown in Eq. (14).

### Our classical algorithm

Comparing the right-hand sides of Eqs. (14) and (31), we conclude that $G\!\left( {U,{s},{t}} \right)$ is equal to the permanent of the matrix $U_{{s},{t}}$, i.e.

(44) \begin{equation*} G\!\left( {U,{s},{t}} \right) = {\text{Perm}}\left( {{U_{{s},{t}}}} \right). \end{equation*}

In other words, with Eqs. (31), (34), and (35), we can express the transition amplitude as the following summation:

(45) \begin{eqnarray*} &&\langle {s_1} \cdots {s_m}|U\!\left| {{t_1} \cdots {t_m}} \right\rangle = \frac{K}{{{d^m}}}\sum \limits _{\left\lbrace z \right\rbrace }\nonumber\\ &&\quad\times {\prod \limits _{k = 1}^m {\big( {\bar{z}_1^{{s_1}}\bar{z}_2^{{s_2}} \cdots \bar{z}_m^{{s_m}}} \big)} } \left( {Uz} \right)_k^{{t_k}} , \end{eqnarray*}

where ${\left( {U {z}} \right)_k} \equiv {u_{k,1}}{z_1} + \cdots + {u_{k,m}}{z_m}$, and the constant is given by $K \equiv v_{s}^2/\prod \nolimits _{k = 1}^m {{{\left( {{s_k}!{t_k}!} \right)}^{1/2}}}$. Note that it is possible to extend our formalism for an arbitrary *m* × *m* matrix *W* = (*w_i, j_*) from the transformation in Eq. (3), which implies that we can define an even more general Glynn estimator,

(46) \begin{equation*} {\rm mGenGly}({z}) \equiv v_{s}^2{\Bigg(\prod \limits _{k = 1}^m {\bar{z}_k^{{s_k}}\Bigg)} \prod \limits _{i = 1}^m \bigg({\sum \limits _{j = 1}^m {{w_{i,j}}{z_j}} }\bigg )^{{t_i}}} , \end{equation*}

which is reduced to the estimator, ${\rm GenGly}({z})$, of Aaronson and Hance for the special cases where *t*_1_ = *t*_2_ =  ⋅ ⋅ ⋅  = *t_m_* = 1, and further reduced to the estimator, ${\rm Gly}({z})$, of Gurvits, when *s*_1_ = *s*_2_ =  ⋅ ⋅ ⋅  = *s_m_* = 1 in addition. An alternative estimator can be found in Huh [52].

We return to the case of quantum optics, where the transformation is necessarily a unitary matrix *U*, with ‖*U*‖ = 1. Now, let us write

(47) \begin{equation*} \langle {s_1} \cdots {s_m}|U\!\!\left| {{t_1} \cdots {t_m}} \right\rangle\,{ =}\, \frac{1}{{{d^m}}}\sum \limits _{\left\lbrace z \right\rbrace } {\frac{{{\text{mGenGly}}\left( {z} \right)}}{{\prod \nolimits _{k = 1}^m {{{({s_k}!{t_k}!)}^{1/2}}} }}} , \end{equation*}

where

(48) \begin{equation*} {\text{mGenGly}}({z}) = v_s^2\Bigg(\prod \limits _{k = 1}^m {\bar{z}_k^{{s_k}}\Bigg)} g\!\left( {z} \right) . \end{equation*}

Here the important point is that we can bound the left-hand side by the following:

(49) \begin{equation*} \left| {{\text{mGenGly}}({z})} \right| \le v_s^2\sqrt{\left( {s_1^{{s_1}} \cdots s_m^{{s_m}}} \right)\left( {t_1^{{t_1}} \cdots t_m^{{t_m}}} \right)} , \end{equation*}

which is due to Eq. (41) and $\left| {\bar{z}_k^{{s_k}}} \right| = s_k^{{s_k/2}}$.

Next, we shall show that one can approximate the transition amplitude with a high probability, by uniformly sampling the more general Glynn’s estimator in Eq. (46), with ${{z}_k} \in \lbrace \sqrt{{s_j}} \, { \omega ^0}, \sqrt{{s_j}} \, {\omega ^1},\ldots , \sqrt{{s_j}} \, {\omega ^{d - 1}}\rbrace ^m$, i.e.

(50) \begin{equation*} \langle {s_1} \cdots {s_m}|U\!\!\left| {{t_1} \cdots {t_m}} \right\rangle \approx \frac{1}{T} \frac{{\sum \nolimits _{k = 1}^T {{\text{mGenGly(}}{{z}_k}{\text{)}}} }}{{\prod \nolimits _{k = 1}^m {{{({s_k}! \, {t_k}!)}^{1/2}}} }} . \end{equation*}

The point is to determine the number of terms *T* for a given error $\epsilon$. Our analysis makes use of the standard Chebyshev bound: for a set of identical, independent, and random variables {*X_i_*}, the probability of taking the average, $\left( {1/T} \right)(\sum \nolimits _{i = 1}^T {{X_i}})$, of *T* variables to deviate from the expectation value, μ = 〈*X_i_*〉, by an amount $\epsilon$ is given by

(51) \begin{equation*} \Pr \left( {\left| {\frac{{\sum \nolimits _{i = 1}^T {{X_i}} }}{T} - \mu } \right| \geqslant \epsilon } \right) \le \frac{{{\sigma ^2}}}{{T{\epsilon ^2}}} , \end{equation*}

where σ^2^ = 〈(*X_i_* − μ)^2^〉 is the variance, which can be bounded by the second moment $\left\langle {X_i^2} \right\rangle$, as ${\sigma ^2} = \left\langle {X_i^2} \right\rangle - {\mu ^2} \le \left\langle {X_i^2} \right\rangle$.

To apply the Chebyshev bound, we identify the transition amplitude as the expectation value

(52) \begin{equation*} \langle {s_1} \cdots {s_m}|U\!\!\left| {{t_1} \cdots {t_m}} \right\rangle \quad \Leftrightarrow \quad \mu , \end{equation*}

and the random variable as follows:

(53) \begin{equation*} \frac{{{\text{mGenGly}}\left( {{{z}_k}} \right)}}{{\prod \nolimits _{k = 1}^m {{{\left( {{s_k}!{t_k}!} \right)}^{1/2}}} }}\quad \Leftrightarrow \quad {X_i} . \end{equation*}

The efficiency of the sampling algorithm depends on the size of the variance; the remaining task is to determine the size of $\left\langle {X_i^2} \right\rangle$, which can be used to bound the size of the variance. From Eq. (49), we have

(54) \begin{equation*} \frac{{| {{\text{mGenGly}}\left( {{{z}_k}} \right)} |}}{{\prod \nolimits _{k = 1}^m {{{\left( {{s_k}!{t_k}!} \right)}^{1/2}}} }} \ \le \ \frac{{{v_{s}}}}{{{v_{t}}}} . \end{equation*}

If ${v_{s}} \gt {v_{t}}$, we can always repeat the argument for the complex conjugate of the transition amplitude, 〈*s*_1_ ⋅ ⋅ ⋅ *s_m_*|*U*|*t*_1_ ⋅ ⋅ ⋅ *t_m_*〉* = 〈*t*_1_ ⋅ ⋅ ⋅ *t_m_*|*U*^†^|*s*_1_ ⋅ ⋅ ⋅ *s_m_*〉, which means that we can always bound the left-hand side by a value smaller than 1, i.e. $\min \lbrace {{v_{s}}/{v_{t}},{v_{t}}/{v_{s}}} \rbrace$.

Consequently, from the Chebyshev bound, by taking a total of $T = O( {1/{\epsilon ^2}} ) \times \min \lbrace {{v_{s}^2}/{v_{t}^2},{v_{t}^2}/{v_{s}^2}} \rbrace$ samples, the error of the approximation in Eq. (50) can be made to be within $\epsilon$ with a high probability close to 1. Note that the evaluation of each sample requires *O*(*m*^2^) steps, as in Eq. (46), the calculation of the summation takes *m* steps and there are *m* factors to multiply. Therefore, the existence of this polynomial-time algorithm, scaling as $O(m^2/\epsilon ^2) \times \min \lbrace {{v_{s}^2}/{v_{t}^2},{v_{t}^2}/{v_{s}^2}} \rbrace$, represents a solution to an open problem raised in the work of Aaronson and Hance [37].

## DISCUSSION

We have presented a general upper bound (Eq. (4)) on the transition amplitudes in sampling bosons for any linear optical network (Eq. (3)). This bound plays the main role in establishing the fact that the computational complexity of decision problems encoded in linear optics cannot be hard problems for classical computers, when the decision problems are encoded in a transition amplitude. However, it may still be possible to encode decision problems in different ways, e.g. problems that may involve many (exponentially) more such amplitudes. For those cases, a direct application of our algorithm may become inefficient.

Nevertheless, this bound yields many implications in quantum physics and computational complexity. The crucial step in proving the bound involves a general theorem (see Eq. (21)) that makes it possible to convert any vacuum-to-vacuum transition amplitude, for some polynomial functions of the boson operators, into a sum of discrete random variables (Eq. (23)). In addition to boson sampling, this theorem is applicable to sampling problems of spin systems [26] and the calculation of elements of the S-matrix in quantum electrodynamics [46].

The connection between the transition amplitudes and the permanents makes it possible to bound the absolute value of the corresponding permanents of matrices (Eq. (17)). Moreover, the classical algorithm proposed by Gurvits [53] can be extended (Eq. (50)) with our bound; the existence of such an algorithm implies that the open problem of Aaronson and Hance in Ref. [37] can now be settled. However, we note that our goal is to estimate the size of the amplitude (probability), a task known as strong simulation, instead of weak sampling where the target distribution is output. The connection between strong and weak simulation has been thoroughly studied in the context of (extended) Clifford circuits [47,48]. Perhaps a similar analysis can also be carried out in the context of linear optics.

Finally, we note that it is straightforward to show that our bound can also be applied to generalize the de-randomizing algorithm for approximating permanents of non-negative matrices, which was discussed by Aaronson and Hance [37].

## Supplementary Material

nwz048_Supplemental_File
