# Supplementary material for: Universal bound on sampling bosons in linear optics and its computational implications
Source: Natl Sci Rev. 2019 Apr 9;6(4):719–29. doi: 10.1093/nsr/nwz048 (PMC8291458; doi:10.1093/nsr/nwz048)
Supplement: nwz048_Supplemental_File [file nwz048_supplemental_file.pdf]

Supplementary materials: Universal Bound on Sampling Bosons in Linear Optics and Its Computational Implications

Man-Hong Yung,[1,2,3,*] Xun Gao,[4] and Joonsuk Huh[5,†]

[1]*Institute for Quantum Science and Engineering and Department of Physics, South University of Science and Technology of China, Shenzhen 518055, China*

[2]*Shenzhen Key Laboratory of Quantum Science and Engineering, Southern University of Science and Technology, Shenzhen 518055, China*

[3]*Central Research Institute, Huawei Technologies, Shenzhen 51829, China*

[4]*Center for Quantum Information, Institute for Interdisciplinary Information Sciences, Tsinghua University, Beijing 100084, China*

[5]*Department of Chemistry, Sungkyunkwan University, Suwon 440-746, Korea*

[*] yung@sustc.edu.cn

[†] joonsukhuh@gmail.com

1. BACKGROUND ON COMPLEXITY CLASSES, P, BPP, BQP

Decision problems contain problems that has a definite answer, either yes or no, from an (classical or quantum) algorithm. The time complexity of an algorithm is measured by the number of elementary steps it takes to reach the answer, in the worst-case scenario. Specifically, the class of problems that can be solved by a classical computer with a polynomial number $O(n^k)$ of steps is labeled as "polynomial time" (P). Here n is the size of the input of the problem. These problems are often regarded as "easy" problems for classical computers. It is because, for most of the practical problems in P, the degree of the polynomial k is usually not very large. Traditionally, a more precise definition of P is stated in terms of the language-theoretic framework: a language L (or decision problem) is in the complexity class P, if and only if there exists an algorithm (or classical circuit $C_n(x)$) that runs in a polynomial time such that,

(i) for all $x \in L$, the circuit outputs "yes", i.e., $C_n(x) = 1$, and

(ii) for all $x \notin L$, the circuit outputs "no", i.e., $C_n(x) = 0$.

So far, we have discussed the idea of deterministic computation only; many more problems can be solved efficiently, if we are allowed to use randomness as well, which allows mistakes to occur occasionally. In this way, we might be able to deduce the correct answer by repeating the non-deterministic algorithm multiple times. More precisely, a language L is in the complexity class "bounded-error probabilistic polynomial time" (BPP), if and only if there exists a probabilistic classical circuit $C_n(x)$ that runs in a polynomial time, such that

(i) for all $x \in L$, the circuit outputs "yes" with a probability larger than or equal to $2/3$, i.e., $\Pr\left(C_n\left(x\right) = 1\right) \geqslant 2/3$, and

(ii) for all $x \notin L$, the circuit outputs "no" with a probability less than or equal to $1/3$, i.e., $\Pr\left(C_n\left(x\right) = 0\right) \leqslant 1/3$.

Finally, the class of problems that can be solved efficiently by a quantum computer, namely "bounded-error probabilistic polynomial time" (BQP), can be defined in a similar way. A language L is in BQP if and only if there exists a polynomial-time quantum circuits $Q_n(x)$, which takes n qubits and output 1 bit, such that

(i) for all $x \in L$, $\Pr(Q_n(x) = 1) \geq 2/3$, and

(ii) for all $x \notin L$, $\Pr(Q_n(x) = 1) \leq 1/3$.

2. BACKGROUND ON COMPLEXITY CLASS BQP AND THE LINEAR-OPTICS VERSION

Let us consider a concrete example. Suppose we apply a quantum computer to simulate the time evolution of a many-spin system, initialized in the state $|x_1x_2x_3...\rangle$ with $x_i \in \{0,1\}$. Then, a quantum circuit is applied to simulate the time operator, i.e., $U \approx e^{-iHt}$. At the end, one would be interested in certain observables, e.g., the two-point correlation function between the first two spins, $\langle\sigma_z^1\sigma_z^2\rangle = \langle\psi_t|\,\sigma_z^1\sigma_z^2\,|\psi_t\rangle$. Note that we can always express it in terms of the probabilities, $P\,(a_1a_2) \equiv \sum_{a_3\cdots} |\langle a_1a_2a_3\cdots|\,U\,|000\cdots0\rangle|^2$, where $a_i \in \{0,1\}$, and the summation is over all indices other than a_1 and a_2. In other words, we can express the correlation function in the following way: $\langle\sigma_z^1\sigma_z^2\rangle = P\,(00) + P\,(11) - P\,(01) - P\,(10)$, and can focus on each of them separately.

For example, let us consider $P(00)$ and express it through $P\,(00) = 0.p_1p_2p_3\cdots$ for $p_i \in \{0,1\}$. Here $x = x_1x_2x_3\cdots$ represents one of the possible inputs to the quantum circuit. The language L contains all the the strings such that $p_1 = 1$. The quantum algorithm can then be repeated until error is reduced to a small value. Then, the whole procedure is repeated for different p_i's, and for other P's. In addition, one may also be interested in the probability of a particular outcome $|y\rangle$. In this case, we have $\Pr\left(Q_n\left(x\right)=1\right) = |\langle y|\,U_x\,|0^{\otimes n}\rangle|^2$.

In the case of quantum optics, the input string x can be represented by the different initial states $|t_1t_2t_3\cdots t_m\rangle$, where $|t_k\rangle \equiv (t_k!)^{-1/2}(a_k^\dagger)^{t_k}\,|\mathrm{vac}\rangle$. The quantum circuit is now replaced with a linear optical circuit, denoted by Q_m^{LO}, which can be decomposed into a polynomial number of the elementary linear-optical components. The unitary matrix associated with the optical circuit is denoted by U_x^{LO}. Furthermore, the language L now contains all of the strings such that the optical circuit outputs specific state $|s_1s_2s_3\cdots s_m\rangle$, which means that, we are interested in the following probability, $\Pr\left(Q_m^{\mathrm{LO}}\left(x\right)=1\right) = \left|\langle s_1s_2\cdots s_m|\,U_x^{\mathrm{LO}}\,|t_1t_2\cdots t_m\rangle\right|^2$. One of the main goals of this work is to show that such a probability can in fact be calculated through a classical non-deterministic algorithm, making the decision problems for linear optics a problem in BPP.

3. BACKGROUND ON ERRORS IN DECISION VERSUS SAMPLING PROBLEMS

We have demonstrated that decision problems can be associated with the measurements of certain observables. In general, one may just need to know the value of these observables to a certain accuracy, not depending on the size of the value. This kind of error is called additive error. An estimation A_{est} of a quantity A to within an additive error ε means that $A-\varepsilon \leqslant A_{\text{est}} \leqslant A+\varepsilon$.

Sampling problems are very different from decision problems. Each sampling problem is associated with a probability distribution D_x for an input x. A sampling problem is solved by an algorithm, if it can produce an approximating distribution D_x^{est} such that $\|D_x^{\text{est}} - D_x\| \leqslant \varepsilon$, where $\|f\| = \sum_i |f_i|$. In particular, $\|D\| = 1$. As the number of D_x is exponentially many, 2^n for n-bit strings, typically, the values of each member in D_x is exponentially small. If one is satisfied only with an additive error with an algorithm, it may just output zero, which tells us nothing. In this case, we will need to increase the accuracy for each probability; instead of additive error, one requires an accuracy within a multiplicative error. An estimation A_{est} of a quantity A to within an multiplicative error means that $A\,(1-\varepsilon) \leqslant A_{\text{est}} \leqslant A\,(1+\varepsilon)$, or $|A_{\text{est}} - A| \leqslant A\varepsilon$. Consequently, we have $\|D_x^{\text{est}} - D_x\| \leqslant \|D_x\|\,\varepsilon = \varepsilon$ and the sampling problem can be solved with such accuracy.

In this work, we are not interested in the sampling problem for linear optics, as boson sampling is now widely regarded as a computationally-hard problem. Instead, we are interested in knowing if the decision problems, to within additive errors, of linear optics is still hard. Our results suggest that there exist a polynomial-time classical algorithm for solving the decisions problems encoded in linear optics.

4. BACKGROUND ON ESTIMATORS

It is known that any $m \times m$ matrix $W = (w_{i,j})$ permanent can be calculated exactly with a scaling $O(m^2\, 2^m)$ using Ryser's formula. Glynn suggested a different algorithm requiring a similar computational cost that the Glynn's formula is given as a normalized form:

$$\text{Perm}\,(W) = 2^{-m} \sum_{\boldsymbol{x}} \text{Gly}(\boldsymbol{x}) \;, \tag{1}$$

where $\text{Gly}(\boldsymbol{x}) \equiv x_1 \cdots x_m \prod_{i=1}^{m} (w_{i,1}x_1 + ... + w_{i,m}x_m)$ is Glynn's estimator, and $\boldsymbol{x} = (x_1, x_2, ..., x_m) \in \{\pm 1\}^m$. Based on the Glynn's formula, Gurvits proposed a polynomial-time randomized algorithm to produce an approximation Perm (W) to the value of the permanent, with an additive error $\pm\epsilon\,\|W\|^m$, i.e.,

$$|\tilde{\text{Perm}}\,(W) - \text{Perm}\,(W)| \leq \epsilon\,\|W\|^m \;, \tag{2}$$

where $\|W\| \equiv \sup_{\boldsymbol{v}\neq 0} \|W\boldsymbol{v}\| \,/\, \|\boldsymbol{v}\|$.

The main idea of Gurvits is that one can convert Glynn's formula into an expectation value:

$$\text{Perm}\,(W) = \mathbb{E}[x_1 \cdots x_m \prod_{i=1}^{m} (w_{i,1}x_1 + ... + w_{i,n}x_m)] \;, \tag{3}$$

An approximation of the permanent is obtained by randomly and uniformly picking T strings $\boldsymbol{x}_k \in \{\pm 1\}^m$, for $k = 1, 2, ..., T$, and evaluate the average value:

$$\tilde{\mathrm{P}}\mathrm{erm}\left(W\right) = \frac{1}{T}\sum_{k=1}^{T} \mathsf{Gly}\left(\boldsymbol{x}_k\right) . \tag{4}$$
